# Coordination of WNT signaling and ciliogenesis during odontogenesis by piezo type mechanosensitive ion channel component 1

**DOI:** 10.1038/s41598-019-51381-9

**Published:** 2019-10-14

**Authors:** Aya Miyazaki, Asuna Sugimoto, Keigo Yoshizaki, Keita Kawarabayashi, Kokoro Iwata, Rika Kurogoushi, Takamasa Kitamura, Kunihiro Otsuka, Tomokazu Hasegawa, Yuki Akazawa, Satoshi Fukumoto, Naozumi Ishimaru, Tsutomu Iwamoto

**Affiliations:** 1 Department of Pediatric Dentistry, Institute of Biomedical Sciences, Tokushima University Graduate School, Tokushima, 770-8504 Japan; 2 Section of Orthodontics and Dentofacial Orthopedics, Division of Oral Health, Growth and Development, Faculty of Dental Science, Kyushu University, Fukuoka, 812-8582 Japan; 3 Department of Interdisciplinary Researches for Medicine and Photonics, Institute of Post-LED Photonics, Tokushima University Graduate School, Tokushima, 770-8504 Japan; 4 Division of Pediatric Dentistry, Department of Oral Health and Development Sciences, Tohoku University Graduate School of Dentistry, Sendai, 980-8575 Japan; 5 Department of Oral Molecular Pathology, Institute of Biomedical Sciences, Tokushima University Graduate School, Tokushima, 770-8504 Japan

**Keywords:** Mesenchymal stem cells, Stress signalling

## Abstract

Signal transmission from the mechanical forces to the various intracellular activities is a fundamental process during tissue development. Despite their critical role, the mechanism of mechanical forces in the biological process is poorly understood. In this study, we demonstrated that in the response to hydrostatic pressure (HP), the piezo type mechanosensitive ion channel component 1 (PIEZO1) is a primary mechanosensing receptor for odontoblast differentiation through coordination of the WNT expression and ciliogenesis. In stem cells from human exfoliated deciduous teeth (SHED), HP significantly promoted calcium deposition as well as the expression of odontogenic marker genes, *PANX3* and *DSPP*, and WNT related-genes including *WNT5b* and *WNT16*, whereas HP inhibited cell proliferation and enhanced primary cilia expression. WNT signaling inhibitor XAV939 and primary cilia inhibitor chloral hydrate blocked the HP-induced calcium deposition. The PIEZO1 activator Yoda1 inhibited cell proliferation but induced ciliogenesis and *WNT16* expression. Interestingly, HP and Yoda1 promoted nuclear translocation of RUNX2, whereas siRNA-mediated silencing of PIEZO1 decreased HP-induced nuclear translocation of RUNX2. Taken together, these results suggest that PIEZO1 functions as a mechanotransducer that connects HP signal to the intracellular signalings during odontoblast differentiation.

## Introduction

Mechanotransduction is one of the mechanisms by which cells sense the extracellular mechanical stimuli and convert them into intracellular biological signals. This form of sensory communication plays important roles not only in various physiological responses including proprioception, touch, balance, and hearing but also in the fundamental biological functions during organ development, tissue homeostasis, and disease conditions^1^. In multicellualar organisms, cells are surrounded by the extracellular fluid that is called tissue interstitial fluid. Therefore, the hydrostatic pressure (HP) via the tissue interstitial fluid is considered to be an important mechanical force in cells. However, the underlying mechanism of HP on odontogenesis has not yet established.

The tooth is a specialized tissue that is composed of three hard tissues, enamel, dentin and cementum, and soft tissue of the dental pulp. Tooth development is initiated by epithelial-mesenchymal interaction that mediates invagination of the oral epithelium-derived odontogenic epithelium into the condensed neural crest-derived ectomesenchyme, which results in the formation of tooth bud^2^. Odontogenic epithelial cells form into the enamel at the crown and cementum at the root. On the other hand, ectodermal mesenchymal cells are responsible for the dentin, the most abundant mineralized tissue in tooth. During dentin formation, the ectodermal mesenchymal cells form into a dental papilla. The peripheral dental papilla cells differentiate into preodontoblasts, creating a contiguous monolayer and expressing a gap junction protein pannexin 3 (PANX3)^3^. Then, the preodontoblasts differentiate into odontoblasts that secrete collagenous and non-collagenous proteins, including dentin sialophosphoprotein (DSPP) to the enamel side while receding to the dental pulp side^4^. The deposition of the initial dentin matrix leads to the formation of predentin, which subsequently mineralizes to form a dentin structure^5^. In this process, runt-related transcription factor 2 (RUNX2) is considered to be an essential transcription factor that regulates the differentiation of dental papilla cells to odontoblasts^6,7^.

Wnt signaling pathway is an important signal cascade that regulates cell fate determination during development, regeneration, and disease^8^. In tooth, several of the Wnt family members, including Wnt3, Wnt3a, Wnt4, Wnt5a, Wnt5b, Wnt6, Wnt7a, Wnt7b, Wnt10a, and Wnt10b, are identified in dental epithelial and mesenchyme during tooth development^9,10^. The canonical Wnt signaling pathway depends on β-catenin and is involved in critical morphogenetic signaling at multiple stages of the tooth patterning and development^11^. The mutation of β-catenin results in the formation of large, misshapen tooth buds and ectopic teeth, whereas the deletion of β-catenin leads to inhibit or arrest of tooth development at the early bud stage^12^. Furthermore, the activation of the canonical Wnt signaling promotes reparative dentin formation^13,14^. Thus, the Wnt signaling pathway plays a crucial role in tooth development and dentin repair^15^.

Almost all eukaryotic cells generate one primary nonmotile cilium that is a specialized protruding structure on the cell surface^16^ and acts as a mechano/chemosensor^17^. The primary cilia are formed in quiescent cells and are generated from centrosomes that act a microtubule-organizing center from the G1 to G2 phase of the cell cycle so that primary cilia play an important role in the inhibition of cell proliferation^16^. Furthermore, abnormal or dysfunctional cilia caused by genetic mutations are associated with ciliopathies that comprise a wide range of symptoms such as primarily retinal degeneration, cerebral anomalies, obesity, and skeletal malformation^18^. Orofaciodigital syndrome (OFD) and Ellis-van Creveld syndrome, with abnormalities in the oral and maxillofacial region and limbs, are craniofacial ciliopathies, and show abnormal tooth development including dentinogenesis imperfecta^19^, indicating that primary cilia may be involved in odontoblast differentiation. Recently, several receptors, including Wnt^20^, sonic hedgehog^21^, Notch^22^, and transforming growth factors (TGFβ)^23^ were identified at the membrane of primary cilia with high density. Thus, primary cilia are also considered to be an essential signaling organelle as an antenna to regulate cell differentiation. However, the molecular mechanisms of ciliogenesis and its role in odontogenesis have not been elucidated.

Piezo type mechanosensitive ion channel component 1 (PIEZO1), also known as FAM38A, is a large transmembrane protein and is conserved among various species^24^. PIEZO1 plays critical roles in the mammalian physiology, including touch, pain sensation, hearing, and blood pressure regulation^25^. Mutations of *PIEZO1* genes have been shown to cause hereditary stomatocytosis^26^. PIEZO1 is also involved in the regulation of neural stem cell differentiation and participates in the mechanosensitive lineage choice of neural stem cells^27^. We have previously reported that PIEZO1 functions as a signal receptor of hydrostatic pressure (HP), and regulates cell fate determination of human mesenchymal stem cell^28^. Furthermore, Piezo1 is expressed in mouse embryonic stem cells and controls cell proliferation^29^. These results suggest that PIEZO1, as a mechanosensing receptor, may play a crucial role in odontogenesis from multipotent stem cells in tooth.

In this study, we demonstrated that HP promotes odontoblasts differentiation and mineralization of multipotent stem cells from human exfoliated deciduous teeth (SHED) through *Wnt16* expression and ciliogenesis, which is mediated by PIEZO1. We also found that both HP and PIEZO1 activator Yoda1 regulate nuclear translocation of runt-related transcription factor 2 (RUNX2) that is a critical transcription factor for odontoblast differentiation. Our results revealed that PIEZO1, a mechanosensing receptor, acts as a conductor to lead a signaling network connecting between mechanical stimuli to chemical signaling in odontoblast differentiation.

## Results

### Sustained HP promotes odontogenic differentiation of SHED

**Figure 1:**
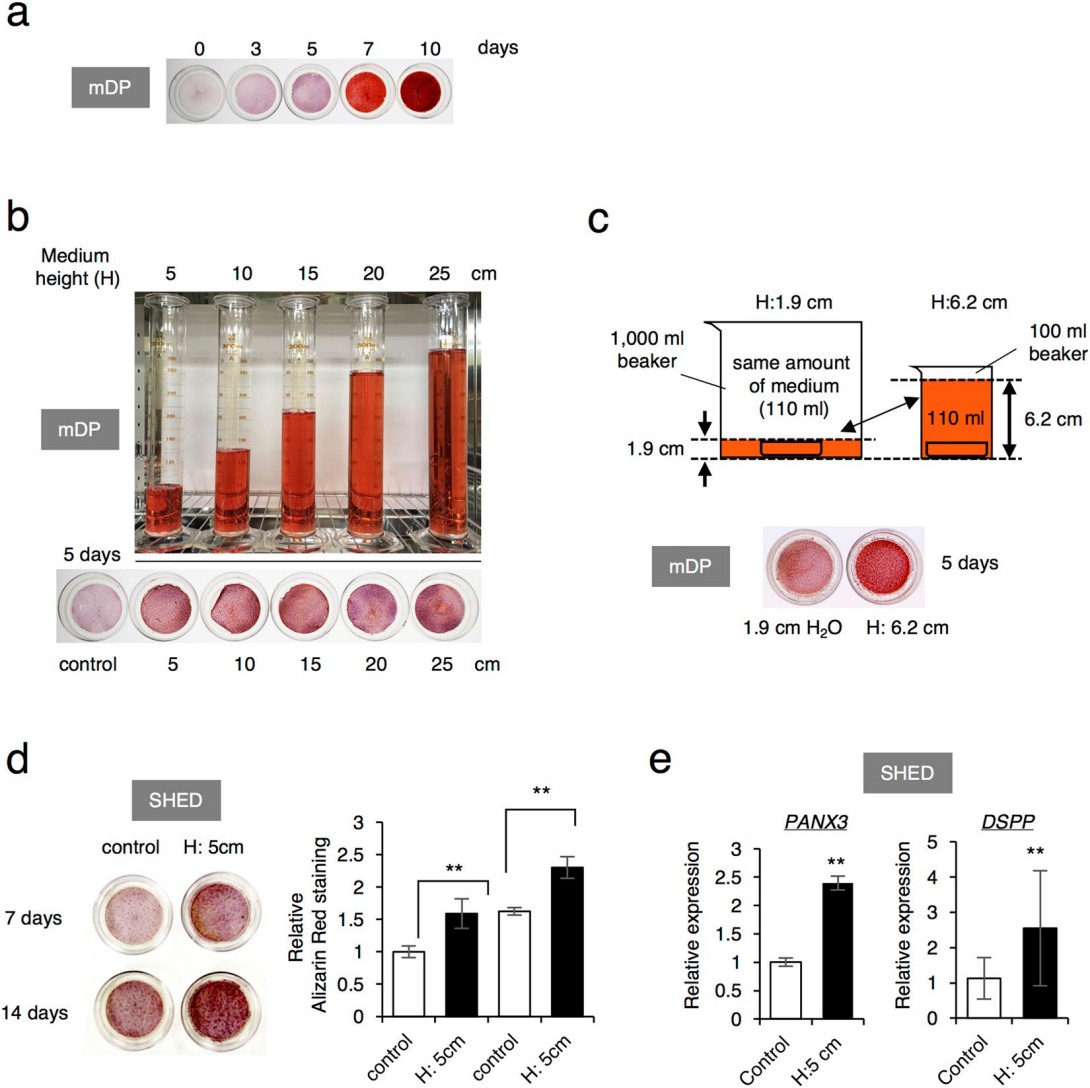
Odontoblast differentiation of SHED in the response to the sustained HP. (**a**) Alizarin Red S staining of the mouse dental mesenchymal cell line mDP cultured with induction (differentiation) medium for 10 days. (**b**) Effects of the sustained hydrostatic pressure (HP) on the differentiation of mDP cells cultured with a different medium height of 5, 10, 15, 20, and 25 cm. Alizarin red S staining was pefomed after 5 days induction. (**c**) A schematic diagram shows the sustained HP methods using the same amount of medium at different medium height. (**d**) Alizarin Red S staining of multipotent stem cells from human exfoliated deciduous teeth (SHED) cultured for 7 and 14 days with or without the HP by the medium height of 5 cm (H: 5cm). The data was representative of 3 independent experiments showed similar results. (**e**) Expression levels of odontogenic marker genes in SHED. Total RNA was extracted after 72 hrs of odontogenic induction with or without the HP by the medium height of 5 cm (H: 5 cm). Gene expression was analyzed by real-time RT-PCR. The data were pooled from three independent experiments. The error bars indicate standard deviations. Statistical analysis was performed using analysis of variance (***p < 0.01).

Mouse odontogenic cell line, mDP, is derived from dental pulp and has the potential to differentiate and mineralize *in vitro*. We cultured these cells in calcification induction media for 10 days and demonstrated their mineralization capacity by staining with Alizarin red S (Fig. 1a). Using this cell line, we first tested whether sustained hydrostatic pressure (HP) affects the mineralization of mDP. To analyze the effect of HP loading on cell culture, we employed the cell culture system where the 35 mm cell culture dish was placed at the bottom of a sterilized syringe and then the media was applied for different heights ranging from 5 to 25 cm (Fig. 1b). Interestingly, we found that at 5 days of culture, the cells exposed to HP loading by the medium height of 5 cm or more showed stronger Alizarin Red S staining than control cells exposed to HP loading by the medium height of 0.3 cm with 2 ml of media (usually an average height for media) (Fig. 1b). Since each dish was cultured with the different volumes of the medium, those differences might affect the promotion of mineralization in the results. Therefore, to clarify whether this result was induced by differences in HP or by differences in the medium volume, the 35 mm cell culture dish was placed in a sterilized 100 ml or 1,000 ml beaker containing the same amount of medium and cultured for 5 days (Fig. 1c). The result showed that cells cultured with the higher level of the medium increased the Alizarin Red S staining when compared to cells with a lower level of medium (Fig. 1c). Thus, we attribute this to the higher HP due to liquid levels of the medium. Therefore, in the following experiments of sustained HP loading, a 35 mm cell culture dish was placed at the bottom of a 100 ml beaker with a medium height of 5 cm.

To confirm the results of accelerated mineralization by HP loading in mDP cells, multipotent stem cells from human exfoliated deciduous teeth (SHED) were cultured with HP. Alizarin Red S staining revealed that HP promoted mineralization of SHED (Fig. 1d). Furthermore, in SHED, the gene expressions of Pannexin 3 (*PANX3*), a pre-odontoblast marker3, and dentin sialophosphoprotein (*DSPP*), a mature odontoblast marker4, was significantly increased by HP (Fig. 1e), indicating that sustained HP promoted cell differentiation of SHED to odontoblasts.

### Sustained HP inhibits cell proliferation and induces ciliogenesis in SHED

**Figure 2:**
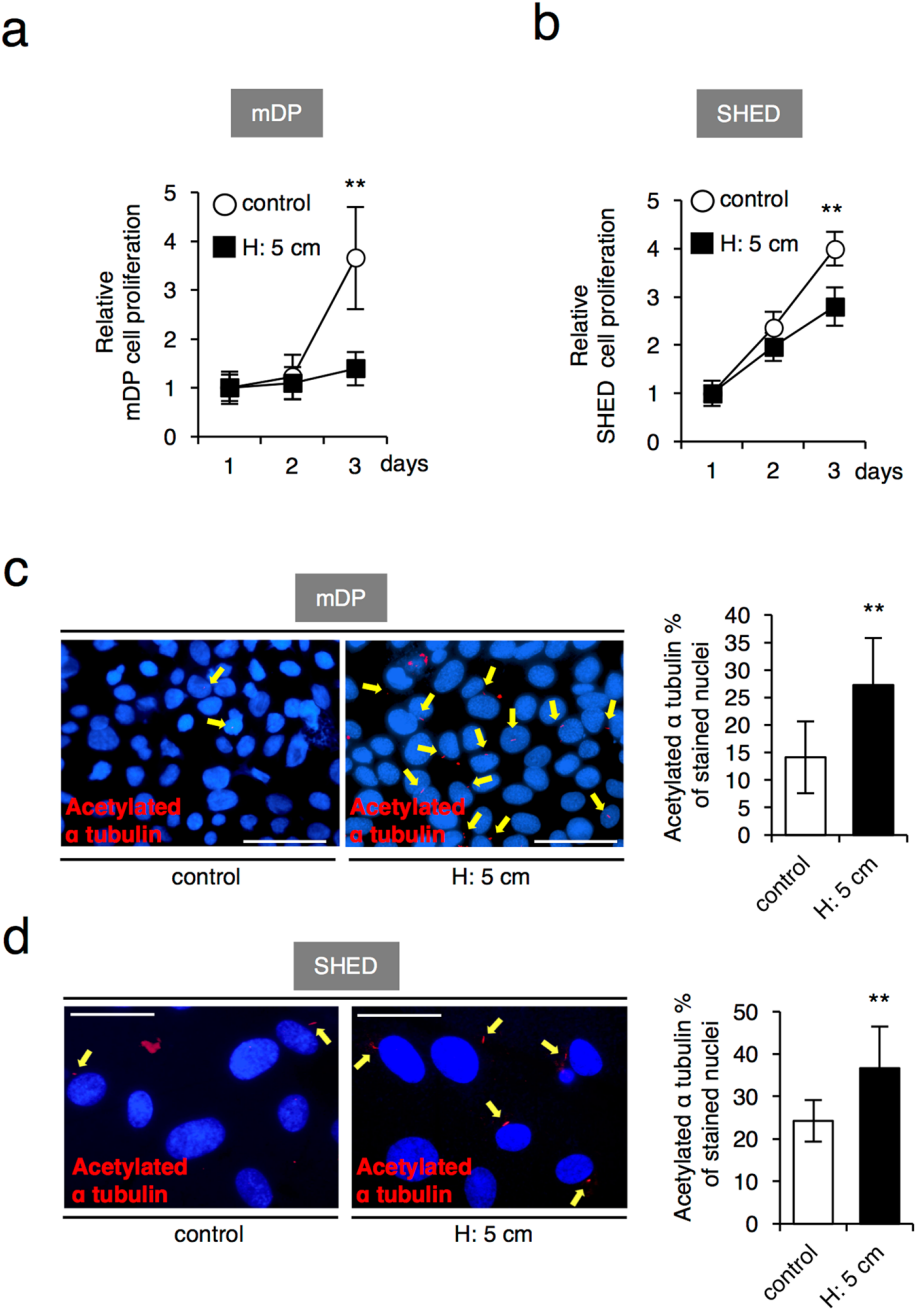
Sustained HP inhibits cell proliferation and induces ciliogenesis in SHED. (**a**) Cell proliferation analysis by a cell counting method in mDP cells (**a**) and SHED (**b**). The cells were cultured with or without the HP by the medium height of 5 cm (H: 5cm) for 24, 48 and 72 hrs. (**c**,**d**) Immunostaining of acetylated α-tubulin in mDP cells (**c**), Scale bar: 50 μm; and SHED (**d**), Scale bar: 25 μm. The cells were cultured with or without the HP by the medium height of 5 cm (H: 5 cm) for 6 hrs, and then immunostaining was perfomed for acetylated α-tubulin. The acetylated α-tubulin-positive cells were counted in twenty randomly selected fields of view under an inverted microscope (20X magnification). The bar graph shows the percentage of α-tubulin-positive cells of total nuclear-stained cells. Red, acetylated α-tubulin; blue, DAPI. The data presented in (**a**,**b**) is a representative of three independent experiments showed similar results. (**c**,**d**) Represent the mean (±standard deviation, SD) of three independent experiments, and each performed in triplicate. Statistical analysis was performed using analysis of variance (**p < 0.01).

To examine the effect of HP on cell proliferation, mDP cells and SHED were cultured with or without HP for 3 days. The cell proliferation was assessed by cell counting method. The results indicate that HP significantly inhibited the cell proliferation of mDP cells (Fig. 2a) and SHED (Fig. 2b). Next, because primary cilia are derived from centrosomes and play a role in blocking cell division^16,17^, we examined whether HP induces primary cilia expression. Acetylation at the ε-amino group of K40 of α-tubulin is enriched in primary cilia and is used a cilia marker protein^30^. We found that HP markedly increased the number of acetylated α-tubulin positive cells in mDP (Fig. 2c) and SHED (Fig. 2d). These results suggest that the inhibition of cell proliferation by HP correlates with HP-induced ciliogenesis.

### The primary cilium is essential for sustained HP-induced mineralization in SHED

**Figure 3:**
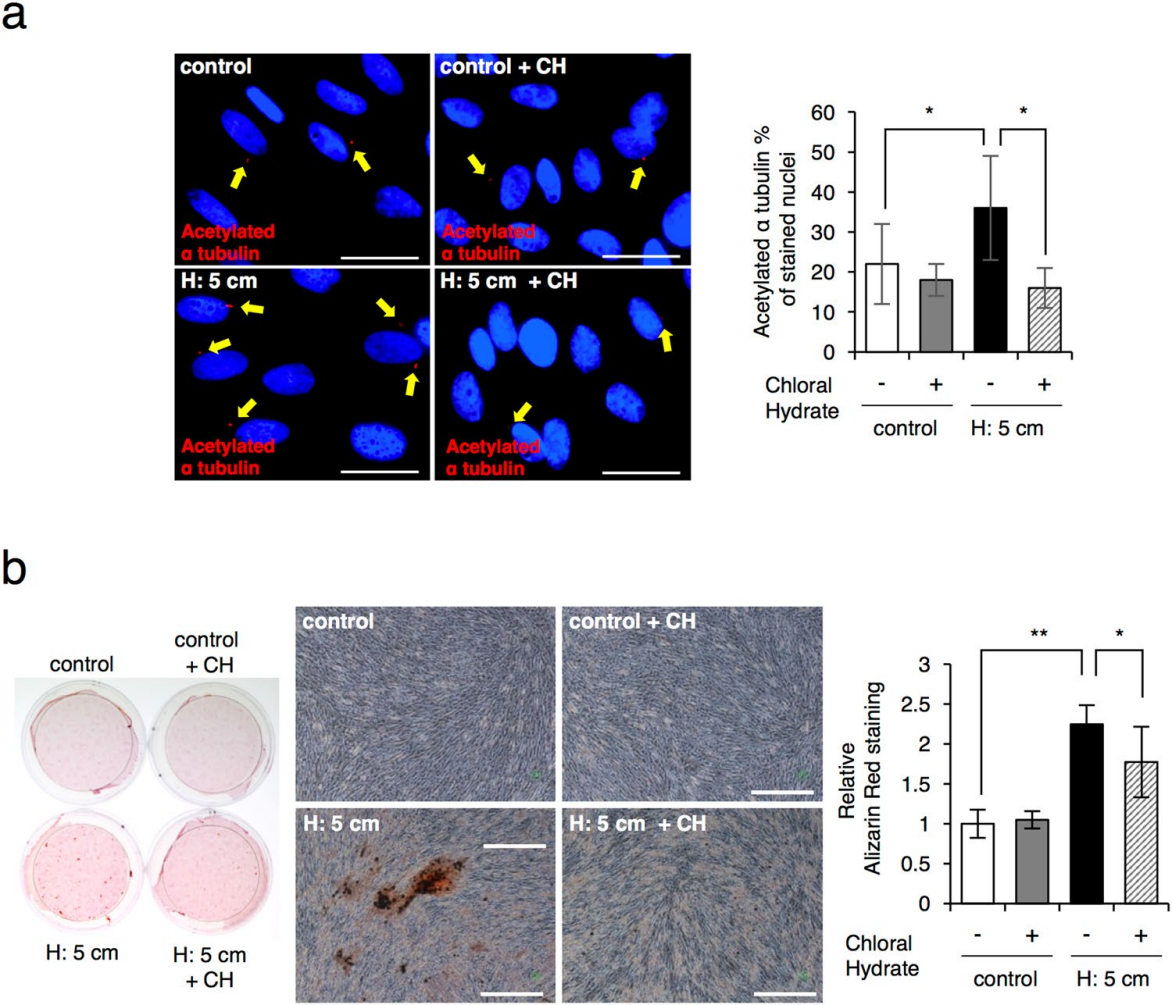
Ciliogenesis is essential for HP induced mineralizeation in SHED. (**a**) Immunostaining and quantification of cilia formation. The SHED were cultured in the presence or absence of 2 μM chloral hydrate (CH) and with or without the HP by the medium height of 5 cm (H: 5cm) for 6 hrs. The acetylated α-tubulin-positive cells were counted in twenty randomly selected fields of view under an inverted microscope (20X magnification). The bar graph shows the percentage of acetylated α-tubulin-positive cells from total cells with nuclear staining. Scale bar: 25 μm. (**b**) Alizarin Red S staining for odontoblast differentiation in SHED cultured in induction media and 2 μM chloral hydrate (CH) for 7 days. Cells were also subjected with or without the HP by the medium height of 5 cm (H: 5 cm). The Alizarin Red-positive areas were quantified using ImageJ. Scale bar, 150 μm. The data, as shown, are the representative of three independent experiments with similar results, and error bars indicate standard deviations. Statistical analysis was performed using analysis of variance.

To test whether the HP-induced primary cilia expression is also required for accelerated mineralization of SHED, we treated SHED cells with chloral hydrate (CH) to inhibit primary cilia expression^31^. We showed that HP-induced primary cilia expression was inhibited by the treatment of 2 μM CH (Fig. 3a). Also, HP-induced calcium deposition decreased in CH-treated cells after 7 days of culture in differentiation media (Fig. 3b). These results suggest that primary cilia expression is involved in the HP-induced mineralization of SHED.

### PIEZO1 is a primary mechanosensing receptor and regulates ciliogenesis in SHED

**Figure 4:**
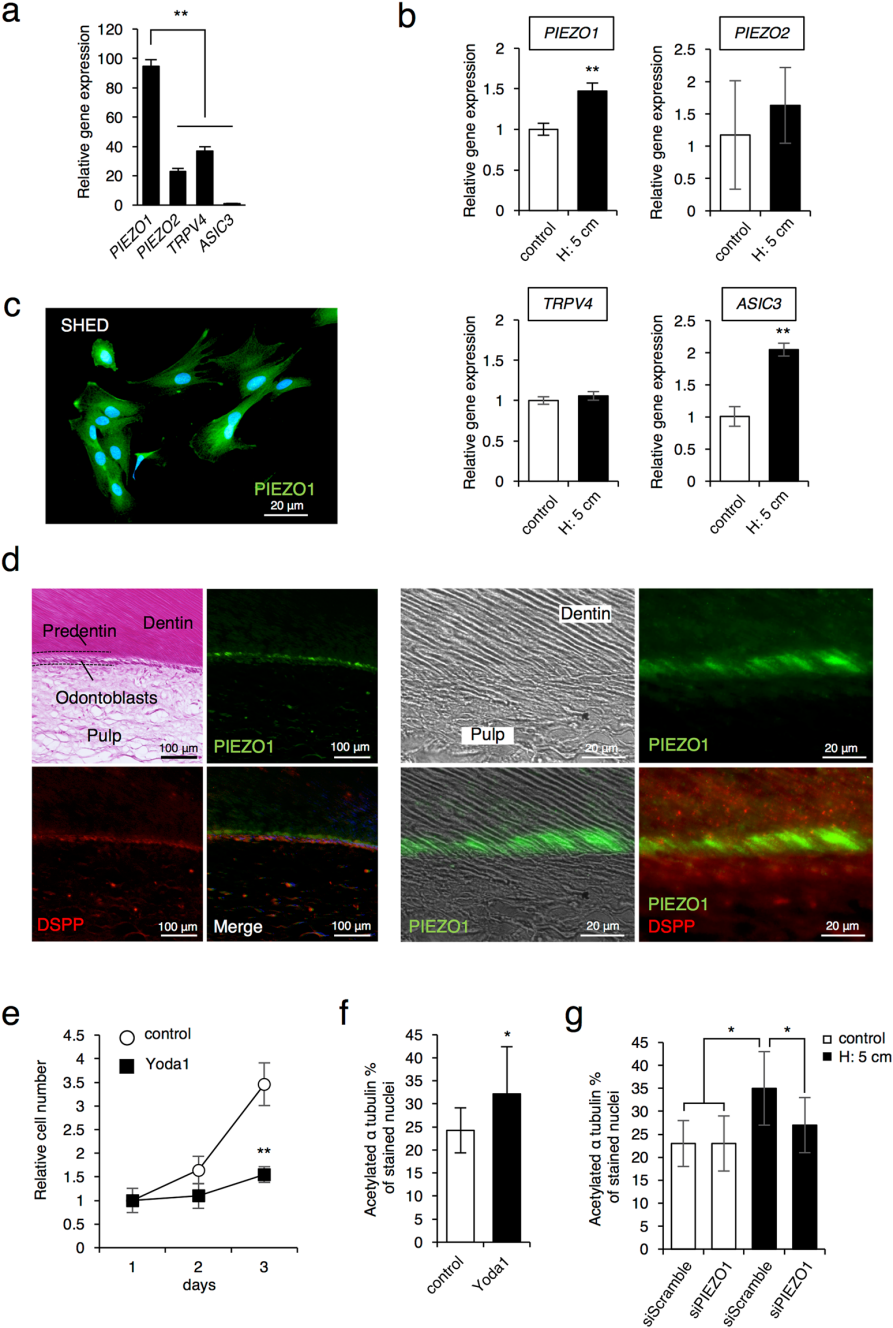
PIEZO1 is primary mechanosensing recepor in SHED and odontoblasts. (**a**) Expression of mechanosensing receptors in SHED was examined by real-time RT-PCR. (**b**) Effect of the the HP by the medium height of 5 cm (H: 5 cm) on the expression of *PIEZO1*, *PIEZO2*, *TRPV4*, and *ASIC3*. SHED cells were cultured with osteogenic differentiation medium at 5 cm medium height (H: 5cm) for 24 hrs. The gene expression was examined by real-time RT-PCR. (**c)** Cellular localizations of PIEZO1 in SHED was determined by immunostaining. Green, PIEZO1; blue, DAPI; Scale bars, 20 μm. (**d**) Immunohistochemistry studies in the tooth section using PIEZO1 and DSPP antibodies. Green, PIEZO1; Red, DSPP, a marker of odontoblasts; blue, DAPI; Scale bars, 100 μm and 20 μm. (**e**) Effect of Yoda1 treatment on cell proliferation. SHED were culutured with or without 5 μM Yoda1 for 24, 48, and 72 hrs, and then cell proliferaiton was assessed by a cell counting method. (**f**) Effect of Yoda1 treatment on cilia formation. SHED cells were cultured with or without 5 μM Yoda1 for 6 hrs, and acetylated α-tubulin-positive cells were counted in twenty randomly selected fields of view under an inverted microscope (20X magnification). The bar graph shows the percentage of acetylated α-tubulin-expressing cells. (**g**) Effect of *PIEZO1*-specific siRNA transfection on cilia formation. SHED cells were transfected with scramble siRNA or *PIEZO1*-specific siRNA for 6 hrs and then cultured with or without the HP by the medium height of 5 cm (H: 5cm) for 6 hrs. The acetylated α-tubulin-positive cells were counted in twenty randomly selected fields of view under an inverted microscope (20X magnification). The bar graph shows the percentage of acetylated α-tubulin-expressing cells. The data presented in (**a**,**b**,**e**) are the representative of three independent experiments showed similar results. For the analyses, the represented in (**f**,**g**) show the data pooled from three independent experiments. The error bars indicate standard deviations. Statistical analysis was performed using analysis of variance (*p < 0.05, **p < 0.01).

Our findings thus suggest an important role of primary cilia in cell proliferation and differentiation of SHED during HP loading. However, the mechanism of the ciliogenesis by HP loading is not known. Therefore, we analyzed the expression of mechanosensing receptors in SHED to identify the initial response of the receptors to HP. Quantitative gene expression analysis showed that among mechanosensitive receptors tested, piezo type mechanosensitive ion channel component 1 (*PIEZO1*) strongly expresses in SHED (Fig. 4a). Furthermore, we also found that after 24 hrs of HP loading, the *PIEZO1* expression significantly increased (Fig. 4b). Although the expression of ASIC3 was also increased by HP loading, its expression level was low compared to the PIEZO1 expression. Thus, PIEZO1 may function as a primary mechanosensing receptor in SHED. Immunostaining analysis using a PIEZO1 antibody in SHED cells showed that PIEZO1 was localized in the plasma membranes and highly enriched at the cellular process (Fig. 4c). Furthermore, to analyze the expression of PIEZO1 in the human tooth, we performed immunohistochemistry analysis on tissue sections prepared from the extracted tooth, we observed a strong signal of PIEZO1 staining in the process of odontoblasts within predentin (Fig. 4d). These results suggest that PIEZO1 is a mechanosensing receptor in SHED and odontoblasts, and may function in cellular processes in response to extracellular stimuli during tooth development and dentin repair.

To elucidate the function of PIEZO1 in SHED, we used a recently identified novel specific PIEZO1 agonist, Yoda1^28,32,33^. SHED were treated with 5 μM Yoda1 for 3 days, and cell proliferation was determined by a cell counting method. We showed that the number of cells significantly decreased in Yoda1-treated cells when compared the dimethyl sulfoxide (DMSO)-treated control cells (Fig. 4e). Also, Yoda1-induced primary cilia expression (Fig. 4f), but the suppression of endogenous PIEZO1 expression by PIEZO1 siRNA inhibited HP-induced primary cilia expression (Fig. 4g). These results suggest that PIEZO1 regulates ciliogenesis under HP loading.

### Sustained HP induced mineralization of SHED through modulation of WNT/β-catenin signaling

**Figure 5:**
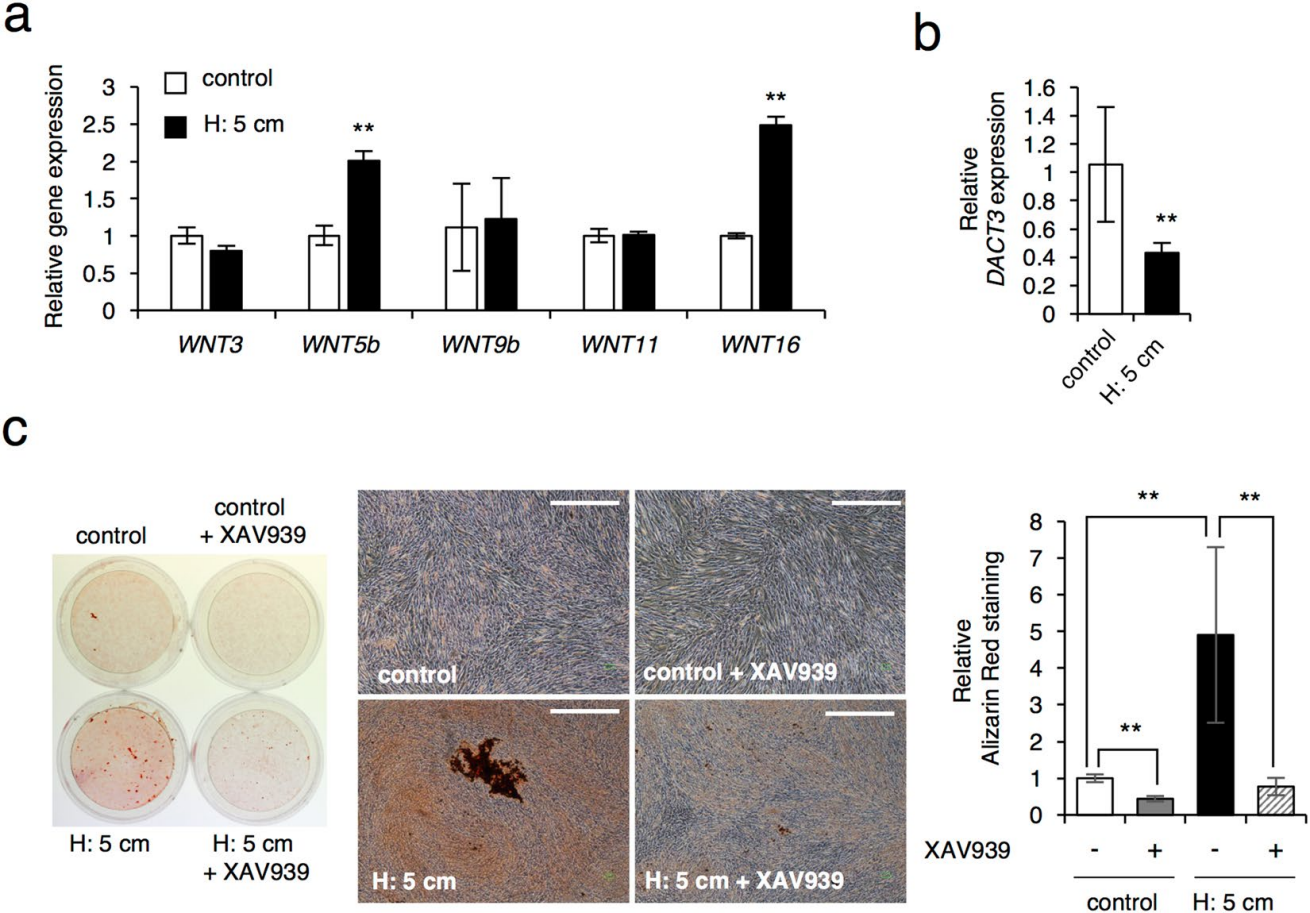
*WNT* expression is essential for HP induced mineralization in SHED. (**a**,**b**) *WNT* and *DACT3* expression in SHED cultured with or without HP by the medium height of 5 cm (H: 5cm) for 24 hrs. Total RNAs prepared from these cells were used for gene expression analysis by real-time RT-PCR for *WNT* (**a**) and *DACT3* (**b**). (**c**) Effect of WNT inhibitor XAV939 on mineralization of SHED by Alizarin Red S staining. SHED were cultured for 7 days in odontogenic induction media with or without 10 μM XAV939 and with or without HP by the medium height of 5 cm (H: 5cm). The Alizarin Red-positive areas were quantified using ImageJ. Scale bar, 150 μm. The data are representative of three independent experiments with similar results, and error bars indicate standard deviations. Statistical analysis was performed using analysis of variance (**p < 0.01).

WNT is a secreted protein that is critically implicated in odontogenesis^8^. Wnt signaling is also mechanosensitive and involves acting downstream of mechanical stimuli in skeletogenesis^34^. Since HP promoted the odontogenic differentiation and mineralization in SHED (Fig. 1d,e), the expression of human WNT genes such as WNT1, 2, 3, 3a, 5a, 5b, 6, 7a, 7b, 8a, 8b, 9b, 10b, 11 and 16, were examined in SHED after culturing with HP for 24 hrs. We found that HP induced the expression of both *WNT5b* and *WNT16* genes (Fig. 5a). There were no significant differences in the expressions of WNT3, 9b, and 11, and no satisfactory amplification products were obtained in others. Furthermore, Dishevelled, a central component of WNT signaling, interacts with dishevelled-binding antagonist of beta-catenin 3 (DACT3), which results in negative regulation of WNT signaling^35^. We found that HP significantly inhibited the *DACT3* expression (Fig. 5b). These results suggest that the induction of mineralization by HP involves the activation of the WNT signaling pathway. Therefore, to confirm whether the WNT signal is necessary for the HP-induced mineralization of SHED, cells were differentiated in the presence of a WNT/β-catenin signaling selectively inhibitor XAV939^36^. XAV939, a tankyrase inhibitor, stabilizes axin by suppressing the poly-ADP-ribosylating enzymes tankyrase 1 and tankyrase 2 and is identified as a selective inhibitor of WNT signaling via β-catenin-mediated transcription^36^. We found that XAV939 markedly inhibited HP-induced mineralization (Fig. 5c), thus suggesting an important role of canonical WNT/β-catenin signal pathway in mineralization of SHED.

### PIEZO1 regulates *WNT16* expression that is involved in mineralization of SHED

**Figure 6:**
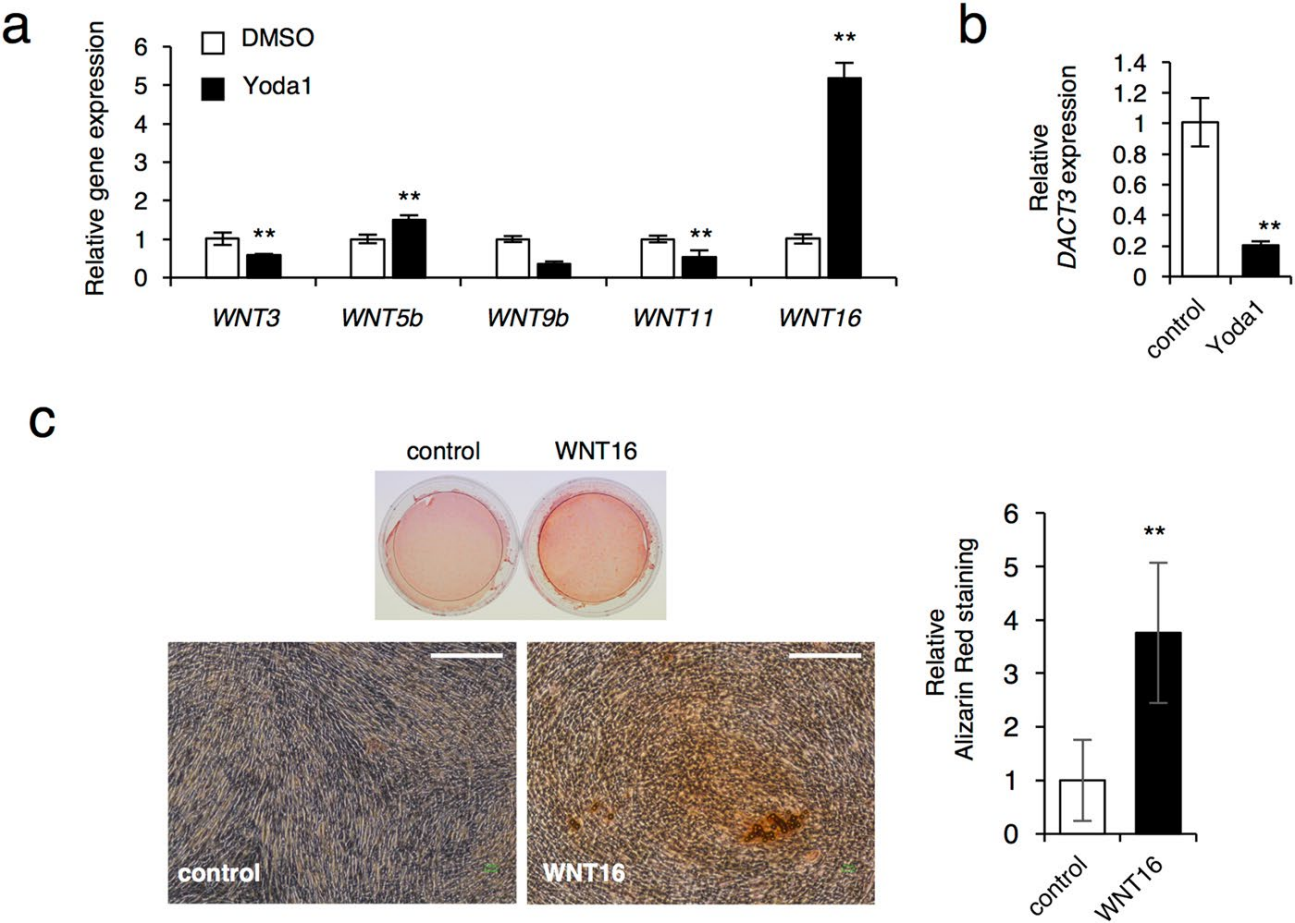
Treatment of Yoda1 upregulates expression of *WNT16* gene which promotes mineralization of SHED. (**a**,**b**) Expression of *WNT* and *DACT3* genes. SHED were cultured with or without 5 μM Yoda1 for 24 hrs. Total RNA was prepared from the cells and analyzed by real-time RT-PCR for *WNT* expression (**a**) and *DACT3* expression (**b**). (**c**) Alizarin Red S staining after WNT16 treatment to SHED. After 7 days of culture of SHED in odontogenic induction media in the presence or absence of 300 ng/ml exogenous WNT16, Alizarin Red staining was performed in SHED. The Alizarin Red-positive areas were quantified using ImageJ. Scale bar, 150 μm. The data, as shown, are representative of three independent experiments with similar results, and error bars indicate standard deviations. Statistical analysis was performed using analysis of variance (**p < 0.01).

Our results, as shown above, thus suggests that PIEZO1 is a primary mechanosensing receptor in SHED (Fig. 4), which led us to hypothesize that PIEZO1 regulates the expression of WNT-related genes. Quantitative gene expression analysis showed that while the PIEZO1 activator Yoda1 markedly induced *WNT16* expression (Fig. 6a), whereas it significantly inhibited the *DACT3* expression (Fig. 6b) when compared with DMSO-treated control. These results are similar to those obtained with HP loading (Fig. 5a,b), suggesting that PIEZO1 plays a crucial role in the regulation of WNT gene expression when exposed to HP. However, no report is yet available to indicate that WNT16 is involved in odontogenesis. Therefore, we examined the role of WNT16 during the mineralization of SHED. We found that WNT16 increased Alizarin red S staining in SHED (Fig. 6c). Taken together, these results suggest that PIEZO1 regulates WNT16 expression, which further promotes the differentiation, maturation, and mineralization of SHED.

### Activation of PIEZO1 induces the nuclear translocation of RUNX2

**Figure 7:**
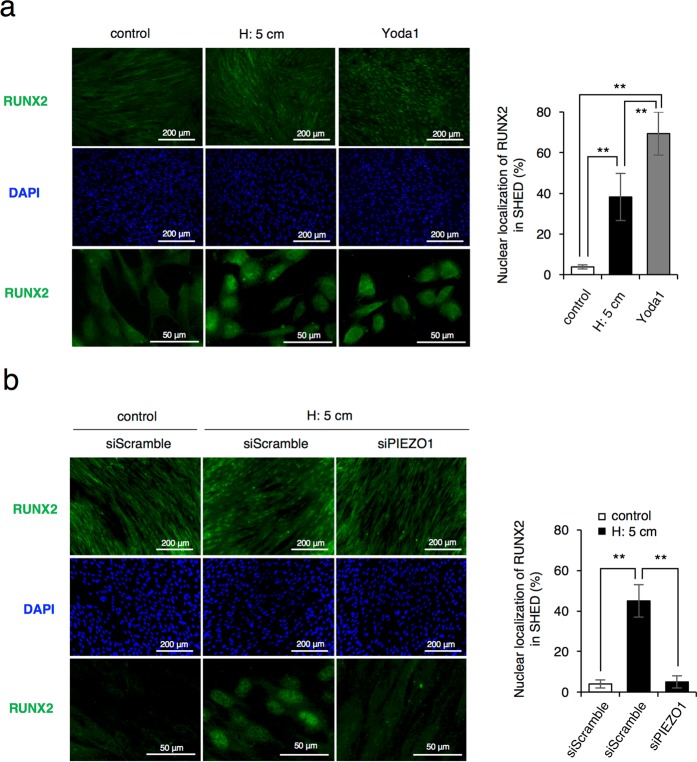
Nuclear translocation of RUNX2 is regulated by the activation of PIEZO1 in SHED. (**a**) Imunostaining analysis for nuclear translocation of RUNX2. SHED were cultured with or without HP by the medium height of 5 cm (H: 5cm) or 5 μM Yoda1 for 24 hrs. Green, RUNX2; blue, DAPI; Scale bars, 200 μm and 50 μm. (**b**) Nuclear translocation of RUNX2 in *PIEZO1*-specific siRNA transfected cells. The *PIEZO1*-specific siRNA transfected cells SHED were replaced and cultured with or without HP by the medium height of 5 cm (H: 5cm) for 24 hrs. RUNX2 nuclear translocation was assessed and quantified as the percentage of the DAPI nuclei-positively stained cells to the total cells. Three different siRNAs specific to *PIEZO1* were tested, and a similar result was obtained. The data are representative of three independent experiments with similar results and error bars indicate standard deviations. Statistical analysis was performed using analysis of variance (**p < 0.01).

Runt-related transcription factor 2 (RUNX2) is a critical transcription factor for osteoblast and odontoblast differentiation^7,37,38^. The activity of the canonical Wnt/β-catenin pathway is modulated by Runx2^39,40^. Therefore, we assessed whether HP loading or Yoda1 affects the intracellular localization of RUNX2. Interestingly, we observed that nuclear translocation of RUNX2 siginificantly increased in cells cultured with HP or Yoda1 (Fig. 7a). On the other hand, down-regulation of PIEZO1 expression by the *PIEZO1*-specific siRNA inhibited the nuclear translocation of RUNX2 in cells cultured with HP (Fig. 7b). These results suggests that under the HP loading, PIEZO1 plays an important role in the regulation of nuclear translocation of RUNX2.

## Discussion

In this study, we demonstrated for the first time that hydrostatic pressure (HP) promotes odontoblast differentiation in multipotent stem cells from human exfoliated deciduous teeth (SHED) through both WNT expression and ciliogenesis that mediated by the piezo type mechanosensitive ion channel component 1 (PIEZO1).

Hydrostatic pressure (HP) via the tissue interstitial fluid involes in an important mechanotransduction in cells. Thus far, the importance of HP on cellular activities has been shown in many experimental studies, but many of them used HP by controlling the gaseous phase with a pressurized special chamber^41^. Previously, we developed a cell culture chamber to control of HP with gaseous phase and found that 0.01MPa (about 75 mmHg) of HP regulates cell fate determination of mesenchymal stem cells^28^. However, it was difficult to observe the effect of HP below 0.005 MPa (about 37.5 mmHg) on the cell culture with this chamber, because a slight change in the amount of air in the sealed chamber during cell culture affects the stability of HP. The mechanism of movement of tissue interstitial fluid is determined by interstitial fluid pressure that is mediated by the difference of the vascular HP and tissue colloid osmotic pressure^42^. The mean value of intracapillary HP is 16–18 mmHg^43^, which acts as a force to push fluid out of the capillaries into the stroma. The average osmotic pressure is approximately 25 mmHg^44^, which acts as a force to pull fluid back from the stroma into the capillaries. The interstitial fluid pressure is considered to be about 1–3 mmHg^45^. Furthermore, the sufficient pressure gradient for the fluid movement was reported to be within about 0.5 mmHg^46^. This exquisite pressure balance suggests that the movement of tissue interstitial fluid in the regulation of cellular activities is physiologically and tightly regulated by a slight change in pressure. Therefore, to observe the effect of the low level of HP on cell differentiation in SHED, the cells were cultured with the modification of the height of the cell culture medium. The HP is determined by an equation: P = r*g*h, where P is the pressure, *r* (rho) is the density of the liquid, *g* is the acceleration of gravity and *h* is the height of the liquid. In cell culture condition, the most remarkable point is that *r* and *g* are the same, so the theoretical HP depends on the height of the cell culture medium. Therefore, based on this theoretical concept, we performed cell culture by changing the height of the medium during culture to observe the effect of the low level of HP. As a result, we found that HP with the height of the cell culture medium at only 5 cm (approximately 3.7 mmHg) promoted the mineralization and the expression of odontogenic marker genes such as *PANX3* and *DSPP* in SHED. These results suggest that applying with the medium at only 5 cm height would be a sufficient mechanical force to promote odontoblast differentiation of multipotent stem cells in tooth.

Primary cilia are considered to be a sensory organelle present in most mammalian cells and act as an antenna in the response to mechano/chemo-stimuli^16,17^. So far, it was shown that the primary cilia formation is regulated by centrosomal and ciliary proteins such as polo-like kinase 4 (Plk4), Cep97, CP110, AurA, and HDAC6^47–49^. Furthermore, in the factors related with the cytoskeleton, actin nucleation-promoting proteins such as cortactin inhibit ciliogenesis^50^. By contrast, actin-severing factors such as cofilin and gelsolin-family proteins promote ciliogenesis^51^. Jasplakinolide (Jasp), a potent inducer of actin polymerization, induced ciliogenesis^52^. These results suggest that the primary cilia formation correlates with mechanotransduction via the cytoskeleton. In this study, we found that among mechanosensing receptors, *PIEZO1* is a preferentially expressed in SHED and induced by HP loading. Furthermore, the strong signal of PIEZO1 was observed in the cellular processes of odontoblasts in tooth. These results suggest that PIEZO1 is a primary mechanosensing receptor in SHED and odontoblast. Interestingly, HP and PIEZO1 activator Yoda1 induced primary cilia expression. Conversely, knockdown of endogenous PIEZO1 expression by siRNA inhibited HP-induced primary cilia expression. These results suggest that PIEZO1 plays a role in the expression of primary cilia. It was reported that PIEZO1 activity was higher in membrane bleb-attached patches that lack cytoskeleton than in cell-attached patches that retain connections between the membrane and cytoskeleton^53^. Furthermore, knocking out filamin, a scaffold protein that binds the actin network to membrane proteins, more activated PIEZO1 in the cell adhesion patch-clamp assay^54^. Thus, PIEZO1 activation would be correlated with the cytoskeleton and scaffold proteins^55,56^, suggesting that the transmission of signals via a mechanosensing receptor PIEZO1 may be involved in the actin polymerization, which results in the promotion of the primary cilia formation. Besides, inhibition of primary cilia formation by chloral hydrate treatment significantly inhibited the HP-induced mineralization in SHED. This result suggests that primary cilia and cilia-related signaling pathways are essential for odontogenesis through the mechanochemical transduction mechanism.

WNT signaling is involved in the signaling pathway in primary cilia and also has an essential role in odontogenesis and dentin repair^15^. Especially, among several types of ciliary-associated signaling pathways, WNT signals are known to be associated with cytoskeleton and cytoskeleton rearrangement^57^. In this study, we found that HP increased the levels of both *WNT5b* and *WNT16* mRNAs. Notably, PIEZO1 activator Yoda1 strongly induced *WNT16* expression. Although the expression and function of WNT16 in tooth were not known, it was reported that WNT16 is associated with cortical bone thickness, cortical porosity, and fracture risk^58–60^. A mouse model with *Wnt16* overexpression in osteoblasts showed an increase of the trabecular bone mass^61^. Besides, Wnt16 and Wnt5a can also directly regulate osteoclast differentiation^62^. Thus, WNT16 plays crucial roles in bone homeostasis. Our findings indicate that exogenously added WNT16 promoted mineralization of SHED. These observations suggest that WNT16 plays a key role in HP- and PIEZO1-mediated mechanical signals for odontoblast differentiation in SHED.

WNT signaling can be divided into two pathways: canonical and non-canonical. The canonical WNT pathway leads to regulation of gene transcription by β-catenin, whereas the non-canonical WNT pathway regulates the cytoskeleton in cell morphology^63^. Several studies have revealed that disruption of ciliated genes leads to aberrant activity in canonical Wnt signaling whilst suppressing non-canonical Wnt signaling^64^. Therefore, ciliogenesis is thought to induce a switch from canonical signaling towards non-canonical wnt signaling^65^. In this study, we found that *DACT3*, a negative regulator of WNT/β-catenin signaling, was dramatically inhibited by both HP and Yoda1. Furthermore, we found that HP-induced mineralization was inhibited by a WNT/β-catenin signaling selectively inhibitor XAV939. These results indicating that canonical WNT/β-catenin signaling pathway may be involved in HP- and PIEZO1-mediated odontoblast differentiation. However, WNT16 can activate both canonical and non-canonical pathways but can regulate to prevent excessive activation of the canonical pathway^66^. WNT signals would play a pivotal role in the complex processes, involving the stop cell division, primary cilia expression, and the beginning of cell differentiation, by the regulation of the switching of canonical to non-canonical pathways. Thus, PIEZO1 may also regulate HP- induced odontoblast differentiation by modulating WNT signaling.

Finally, to delineate the mechanism between chemical and physical factors in the differentiation process, we focused on RUNX2 that is a critical transcription factor for odontogenesis^7,37,38^. We found that HP and Yoda1 promoted the nuclear translocation of RUNX2, but the suppression of endogenous PIEZO1 inhibited it. These results indicate that in the response to HP, PIEZO1 could be a regulator of the nuclear translocation of RUNX2 during odontoblast differentiation. However, studies on developing bone have indicated that Wnt/β-catenin signaling positively regulated RUNX2^67^. Although further studies are needed to elucidate whether the nuclear translocation of RUNX2 is directly regulated by PIEZO1 signaling or indirectly induced by WNT expression, PIEZO1 may participate in RUNX2-WNT signaling in odontoblast differentiation.

In conclusion, we showed that PIEZO1, a mechanosensing receptor, receives and transduces HP signal into the cellular biomolecular signal by modulating WNT signaling and ciliogenesis during odontoblast differentiation. Furthermore, we found that HP with the medium height at only 5 cm promoted mineralization of SHED. SHED are important cell sources for regenerative medicine. Thus, our results provide new insights into the molecular mechanisms of mechanical sensitivity in odontoblast differentiation and also would lead to the development of a new therapeutic approach and a novel cell culture device for regenerative medicine.

## Material and Methods

### Reagents

The Alizarin Red S staining kit was purchased from PG Research (Tokyo, Japan). Yoda1 and XAV939 were purchased from Tocris Bioscience (Bristol, UK) and recombinant human WNT16 protein from R&D systems (Minneapolis, USA). Chloral hydrate was obtained from Tokyo Chemical Industry (Tokyo, Japan).

### Teeth collection

Exfoliated deciduous teeth without caries were obtained from 8 healthy children aged between 8–13 years old (5 males and 3 females), and the written informed consent was obtained from their parent based on the guidelines set by the ethics committee of our hospital (Tokushima, Japan). To obtained stem cells from human exfoliated deciduous teeth (SHED), extracted teeth were immediately placed in α-MEM (Wako, Japan), and were transferred to laboratory within 30 min. Ethical approval was obtained from the Ethics Committee of Tokushima University Hospital (approval no. 1799).

### Isolation of SHED

The teeth were washed with phosphate balanced saline solution (PBS) and then soaked with 2X Antibiotic-Antimycotic (Anti-Anti, Nacalai tesque, Japan) in PBS. The teeth were mechanically broken into pieces with sterile pliers to collect the pulp tissues, which were then disrupted with a acaple blade and digested in a solution with 3 mg/ml collagenase (Wako, Japan) and 4 mg/ml dispase (Wako, Japan) for 1 hr at 37 °C in a CO_2_ incubator. After diluting with α-MEM, the cell suspension was passed through a 40 μm cell strainer (Falcon, USA) and centrifuged at 1,000 rpm for 5 min. The cell pellet was resuspended with α-MEM supplemented with 10% heat-inactivated fetal bovine serum (FBS) and 1% Anti-anti. Single-cell suspensions were cultured in a regular medium as reported^68,69^. Cells at 50–60% confluency were passaged with 3–5 times and then used for the experiments. We successfully isolated SHEDs from six indipendent donors and teeth.

### Cell culture

SHED were maintained in a growth medium containing α-MEM supplemented with 10% FBS and 1% Anti-anti. A dental mesenchymal cell line (mDP) was culutured in DMEM with 10% FBS and 1% Anti-anti at 37 °C in a humidified chamber with 5% CO_2_. For the following experiments, cells were seeded at 4.0 × 10^4^ cells/well on the 35 mm glass bottom dish (Matsunami, Japan) with growth medium one day before the treatment. For hydrostatic pressure (HP) loading, the cell culture dish was placed at the bottom of a syringe or beaker and filled the media to the height of 5, 10, 15, 20, and 25 cm in order to expose the sustained HP. The cells cultured with a 0.3 cm height of media under atmospheric pressure were used as the control. Note that in this study, since atmospheric pressure was regarded as the zero reference, HP means gauge pressure. For odontogenic differentiation, induction (differentiation) media contained growth medium and 10 mM β-glycerophosphate, 150 μg/mL ascorbic acid, and 10^−8^ M dexamethasone, with changing the induction media every 2 days. Calcium deposition, an indicator of mineralization, was determined by Alizarin Red S staining for according to the manufacturer’s protocol. The Alizarin Red S-positive areas were quantified by NIH-ImageJ 1.48v (National Institutes of Health, USA).

### Quantitative RT-PCR

Gene expression was analyzed by real-time RT-PCR. Total RNA was extracted from cultured cells using the TRIzol reagent (Invitrogen) according to the manufacturer’s protocol. Two micrograms of total RNA were used to generate the first-strand cDNA with the PrimeScript RT Master Mix (Perfect Real Time; Takara, Japan). Real-time PCR was carried out using PCR SYBR Premix Ex Taq II (Takara, Japan) and a Thermal Cycler Dice real-time system (Takara, Japan) with the following conditions: 10 s at 95°C, followed by 40 cycles of 95°C for 5 s and 60°C for 30 s, with a final 5 s at 95°C and 30 s at 60°C. For the *DSPP* gene expression, PCR condition was at 10 s at 95°C, followed by 45 cycles of 95°C for 5 s and 63°C for 30 s, with a final 5 s at 95°C and 30 s at 60°C. The reactions were run in triplicate and repeated at least three times. The primers sequence are shown in Supplementary Table S1.

### Cell proliferation

Cell proliferation was determined by a cell counting methods. Cells were plated at 2.0 × 10^3^ cells/well on the 35 mm glass bottom dish and maintained with or without 5 cm H_2_O for 24, 48, and 72 hrs. The total cell numbers were counted in twenty randomly selected fields of view under an inverted microscope with 20X magnification.

### Immunohistochemistry analysis

For the detection of acetylated α-tubulin and PIEZO1, cultured cells were fixed with 4% paraformaldehyde at room temperature (RT) for 5 min. For the detection of RUNX2, cells were fixed with acetone at −20 °C for 3 min. For acetylated α-tubulin and RUNX2 detecions, the fixed cells were permeabilized with 0.2% TritonX-100 for 30 min. Then, the blocking was performed with 2% Bovine Serum Albumin (Sigma-Aldrich) in PBS for 1 h at RT for acetylated α-tubulin detection, or with Universal Blocking Reagent (Biogenex) for 6 min at RT for PIEZO1 and RUNX2 detection. The tooth sections were deparaffinized in xylene and rehydrated with water prior to antigen retrieval by Liberate Antibody Binding Solution (L.A.B. Solution, Polyscience) and then washed with PBS. The sections were incubated with Universal Blocking Reagent for 6 min and then with the primary antibodies. The following antibodies were used for immunohistochemistry: mouse anti-alpha Tubulin (acetyl K40) antibody [6-11B-1] (Abcam), rabbit anti-PIEZO1 antibody (Novus Biologicals), mouse anti-DSPP antibody (Santa Cruz Biotechnology, Germany), mouse anti-Runx2 (Cbfa1) mAb (Medical & Biological Laboratories). Alexa Fluor 488- or 594- conjugated secondary antibodies (Invitrogen) were used for detecting the primary antibody. Immunofluorescence was analyzed with an Olympus BX50 microscope (Tokyo, Japan).

### siRNA experiments

Cells density with 60–80% confluency were transfected with siRNA using Lipofectamine™ RNAiMAX Transfection Reagent (Invtitrogen) according to the manufacturer’s protocol. The following siRNAs were used: ON-TARGET plus Human PIEZO1 siRNA (J-020870-11, 12, and 13; Dharmacon). ON-TARGET plus siCONTROL non-targeting pool siRNA (D-001810-1005; Dharmacon) was used as a control.

### Statistical analysis

For the analyses represented in Figs 1e, 2c,d, 4f,g, the data were pooled from three independent experiments. In Figs 1d, 2a, 2b, 3, 4a,b,e, 5, 6 and 7, the data presented is representative of 3 independent experiments showed similar results. The error bars indicate standard deviations. Statistical analysis was performed using analysis of variance (*p < 0.05, **p < 0.01).

## Supplementary information

Dataset1

## Data Availability

The datasets are available from the corresponding author on reasonable request.
