# Supplementary material for: Coordination of WNT signaling and ciliogenesis during odontogenesis by piezo type mechanosensitive ion channel component 1
Source: Sci Rep. 2019 Oct 14;9:14762. doi: 10.1038/s41598-019-51381-9 (PMC6791893; doi:10.1038/s41598-019-51381-9)
Supplement: Supplementary file 1 — Dataset1 [file 41598_2019_51381_MOESM1_ESM.pdf]

Coordination of WNT signaling and ciliogenesis during odontogenesis by piezo type mechanosensitive ion channel component 1

Aya Miyazaki[1], Asuna Sugimoto[1], Keigo Yoshizaki[2], Keita Kawarabayashi[1], Kokoro Iwata[1], Rika Kurogoushi[1], Takamasa Kitamura[1], Kunihiro Ohtsuka[3], Tomokazu Hasegawa[1], Yuki Akazawa[1], Satoshi Fukumoto[4], Naozumi Ishimaru[3], Tsutomu Iwamoto[1,*]

Supplementary Table S1

Used primers for PCR

Human primers		Sequence	Product size
PANX3	forward	5'-TGAAGATCCGGCAGAAGAGT-3'	74
	reverse	5'-GTATCGTTCTTTCCGAGCC-3'	
DSPP	forward	5'-AGAGGACACCCAGAAGCTCA-3'	271
	reverse	5'-TTCTGATTTTTGGCCAGGTC-3'	
PIEZO1	forward	5’-CAGGCCTATGAGGAGCTGTC-3’	170
	reverse	5’-TTGTAGAGCTCCCGCTTCAT-3’	
PIEZO2	forward	5’-GCCCAACAAAGCCAGTTGAA-3’	167
	reverse	5’-GGGCTGATGGTCCACAAAGA-3’	
TRPV4	forward	5’-GACGGGGACCTATAGCATCA-3’	228
	reverse	5’-AACAGGTCCAGGAGGAAGGT-3’	
ASIC3	forward	5’-ATGGCGCAACTCTATGCCCGT-3’	202
	reverse	5’-TGATGTCCAGCCCATTGCCCA-3’	
WNT1	forward	5’-ATGGGGCTCTGGGCGCTGTTGC-3’	252
	reverse	5’-CCCACTCACGCTGTGCAGGATC-3'	
WNT2	forward	5’-ATGACCAAGTGTGGGTGTAAGTTC-3'	159
	reverse	5’-TGTAGAAGGGAAGGTGGATGGT-3'	
WNT3	forward	5'-TGCCAGGAGTGTATTCGCATC-3'	110
	reverse	5'-TTCCCATGAGACTTCGCTGA-3'	
WNT3a	forward	5’-AACCGCCACAACAACGAGGCT-3'	306
	reverse	5’-AGGGTTGGGCTCGCAGAAGTT-3'	
WNT5a	forward	5’-TTACCACTGCAACTATTGCACCTC-3'	146
	reverse	5’-CACAATGAACCTTTAGTTTCCAACC-3'	
WNT5b	forward	5'-TTCTGACAGACGCCAACTCC-3'	154
	reverse	5'-GCCATGTGCTCCTGGTACAAT-3'	
WNT6	forward	5’-GACGAGAAGTCGAGGCTCTT-3'	88
	reverse	5’-CCTCGTTGTTGTGCAGTTG-3'	
WNT7a	forward	5’-GCCTGGACGAGTGTCAGTTTCA-3'	276
	reverse	5’-CGATGCCGTAGCGGATGTC-3'	
WNT7b	forward	5’-CACAGAAACTTTCGCAAGTGGA-3'	127
	reverse	5’-GGCCAGGAATCTTGTTGCAG-3'	
WNT8a	forward	5’-TATGCTCTGGGCAGCTCTGG-3'	257
	reverse	5’-CAAGCAAACTGGAACTTGCACTC-3'	
WNT8b	forward	5’-TGGTCCAAAGGCTTACCTGATT-3'	92
	reverse	5’-GGTCCCAGGCAAACTGATACTT-3'	
WNT9b	forward	5'-ATGGAGCGCTGCACCTGTGAT-3'	190
	reverse	5'-TCACAGCCTTGATGCCCACGT-3'	
WNT10b	forward	5’-CATGCATGGACTTTGGAGAG-3'	156
	reverse	5’-TGTGCCATGACACTTGCATTT-3'	
WNT11	forward	5'-AAACAGGATCCCAAGCCAATAAACT-3'	122
	reverse	5'-GATGGAGCAGGAGCCAGACA-3'	
WNT16	forward	5'-AAGTGAAGGCTGGCACTGG-3'	188
	reverse	5'-GGCAGTCTACTGACATCAACTTGG-3'	
DACT3	forward	5'-AACCTTCTGTGGGGACAGTG-3'	178
	reverse	5'-GTAGGGCGCTGAGAAGGAC-3'	
GAPDH	forward	5’-GCACCGTCAAGGCTGAGAAC-3’	138
	reverse	5’-TGGTGAAGACGCCAGTGGA-3’	
